# Anatomical characteristics of mental foramen and canal: A cone-beam computed tomography analysis

**DOI:** 10.4317/jced.61861

**Published:** 2024-08-01

**Authors:** Ebad Mallahi, Farida Abesi, Fatemeh Rajaei-Rad, Hemmat Gholinia

**Affiliations:** 1 Student Research Committee, Babol University of Medical Sciences, Babol, Iran; 2 Dental Materials Research Center, Department of Oral and Maxillofacial Radiology, Dental Faculty, Babol University of Medical Sciences, Babol, Iran; 3 Department of Oral and Maxillofacial Surgery, Dental Faculty, Babol University of Medical Sciences, Babol, Iran; 4 Health Research Institute, Babol University of Medical Sciences, Babol, Iran

## Abstract

**Background:**

So far, different studies have endeavored to evaluate the position and dimensions of mental foramen and canal using cone-beam computed tomography (CBCT) images with various results. This study aimed to assess the anatomical variations of the mental foramen and canal utilizing CBCT images.

**Material and Methods:**

In this retrospective observational study, we investigated CBCT scans of 355 patients (710 terminal branches of mental canal and foramen) who were referred to a private dental and maxillofacial radiology center in Babol, during 2020-2022. We recorded different anatomical variations of mental foramina and canals on left (n=355) and right (n=355) mandibles.

**Results:**

Most of the mental canals had a distal opening (n=334, 47.0%). The distance between the mental foramen and the lower mandibular border was greater on left mandible (13.92±3.73 mm) than on the right mandible (12.25±3.94 mm) (*p*<0.001). On left mandible, the vertical diameter of the mental foramen, as well as the distance between the mental foramen and the lower mandibular border, were significantly greater in men than in women. On right mandible, the distance between the mental foramen and the upper alveolar crest edge, as well as the distance between the mental foramen and the lower mandibular border, were significantly greater in men versus women. Finally, on right mandible, the vertical diameter of the mental foramen was significantly greater in subjects aged >45 years compared with those aged ≤45 (*p*=0.024).

**Conclusions:**

There were notable variations in the morphological characteristics of the mental foramen and canal, which should be considered by clinicians.

** Key words:**Mental foramen, mental canal, cone-beam computed tomography.

## Introduction

The precise position and dimensions of the mental foramen and its associated terminal branch of the canal are crucial for various dental and maxillofacial procedures and pathologies. The mental foramen is a critical anatomical landmark through which the mental nerve and vessels exit. The terminal branch of the canal, originating from the mandibular canal, traverses the mandible and emerges from the mental foramen (1,2). Accurate localization and measurement of these structures are essential to avoid potential nerve damage during surgical procedures. Previous studies have utilized various methods, such as cadaveric dissection, clinical measures, and radiographic techniques, to assess the mental foramen and its associated canal. However, these methods have limitations, including sample size restrictions and potential measurement errors, leading to image distortion and superimposition (3,4).

Cone-beam computed tomography (CBCT) has revolutionized oral and maxillofacial imaging by providing high-resolution, three-dimensional images with minimal distortion. This imaging technique can provide a powerful tool for visualizing and assessing the mental foramen and its associated canal. CBCT can offer images that allow for precise measurements and evaluation of anatomical variations (5-7). So far, different studies have endeavored to evaluate the position and dimensions of mental foramen and canal using CBCT images with various results (8-10).

The objective of the present study was to investigate the anatomical characteristics of the mental foramen and terminal branch of the canal utilizing the CBCT imaging method in Babol, northern Iran. Our findings will pointedly contribute to enhancing the safety and precision of dental and maxillofacial procedures. Additionally, this study will improve patient care and treatment planning by providing clinicians with reliable information on the position and dimensions of these important anatomical structures.

## Material and Methods

-Participants and locations

In this analytical observational study, we retrospectively examined CBCT images of subjects referred to a private dental and maxillofacial radiology center in Babol during 2020-2022. The inclusion criteria were as follows: 1) CBCT images of good quality that included the mental foramen and the surrounding anatomical areas; the location of the mandibular premolars was assessed in these images. 2) The study subjects were aged at least 18 years. The exclusion criteria included: 1) Evidence or history of surgery or trauma in the head and neck region, congenital anomalies of the face, bone diseases, skeletal asymmetry, malignancy, or any type of tumor or lesion in the head and neck region. 2) Absence of premolars in the desired area. 3) Presence of a hidden tooth in the desired area.

CBCT imaging

The CBCT images under examination were obtained using the ACTEON X-MIND® TRIUM ITALY device and Ondemand3D software or the Giano unit device (Newtom, Verona, Italy). The images were acquired in sections of 0.5 mm with intervals of 1 mm. The measurements were performed using NNT software (Newtom, Verona, Italy). All image evaluations occurred in a semi-dark room on a Samsung 21” monitor. The analysis was conducted by both an oral, maxillofacial radiology specialist and a maxillofacial surgery specialist simultaneously. Their findings were recorded as a single opinion in a checklist specifically designed for this purpose. In this checklist, the tooth number (4 or 5) corresponding to the mental foramen area section was also documented. Additionally, in terms of measuring the distance between the mental foramen and the alveolar crest, it was noted whether the measurement was taken from the interdental bone location or the alveolar location of the respective tooth. Given that the mental foramen’s position varied relative to the teeth, measuring it in the interdental or dental area was possible, depending on its specific location. We made sure that the measurements were accurate and reliable.

-Data collection

**Figure 1:**
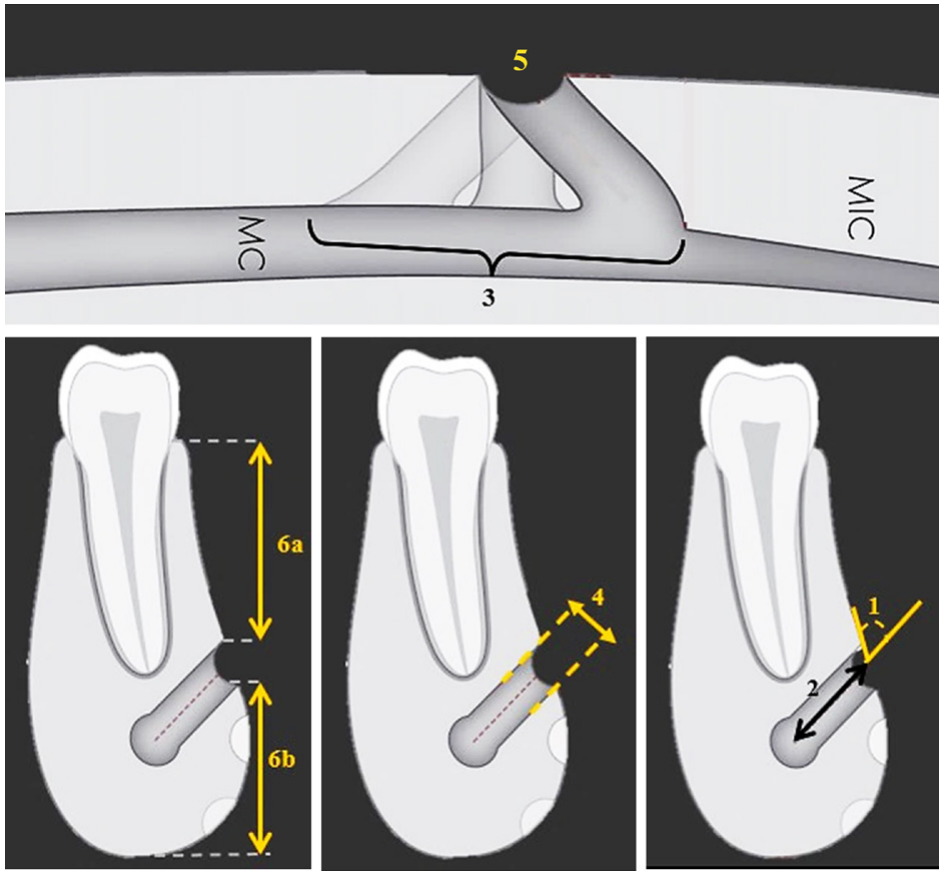
Schematic views of the assessed parameters. Cross-sectional planes included angle of the mental canal (1), length of the mental canal (2), vertical diameter of the mental foramen (4), and distance from mental foramen to the upper edge of the alveolar crest (6a) and to lower mandibular border (6b). Axial planes included opening direction of mental canal (3) and width of the mental foramen (5). Abbreviations: MC, mandibular canal; MIC, mandibular incisive canal.

**Figure 2:**
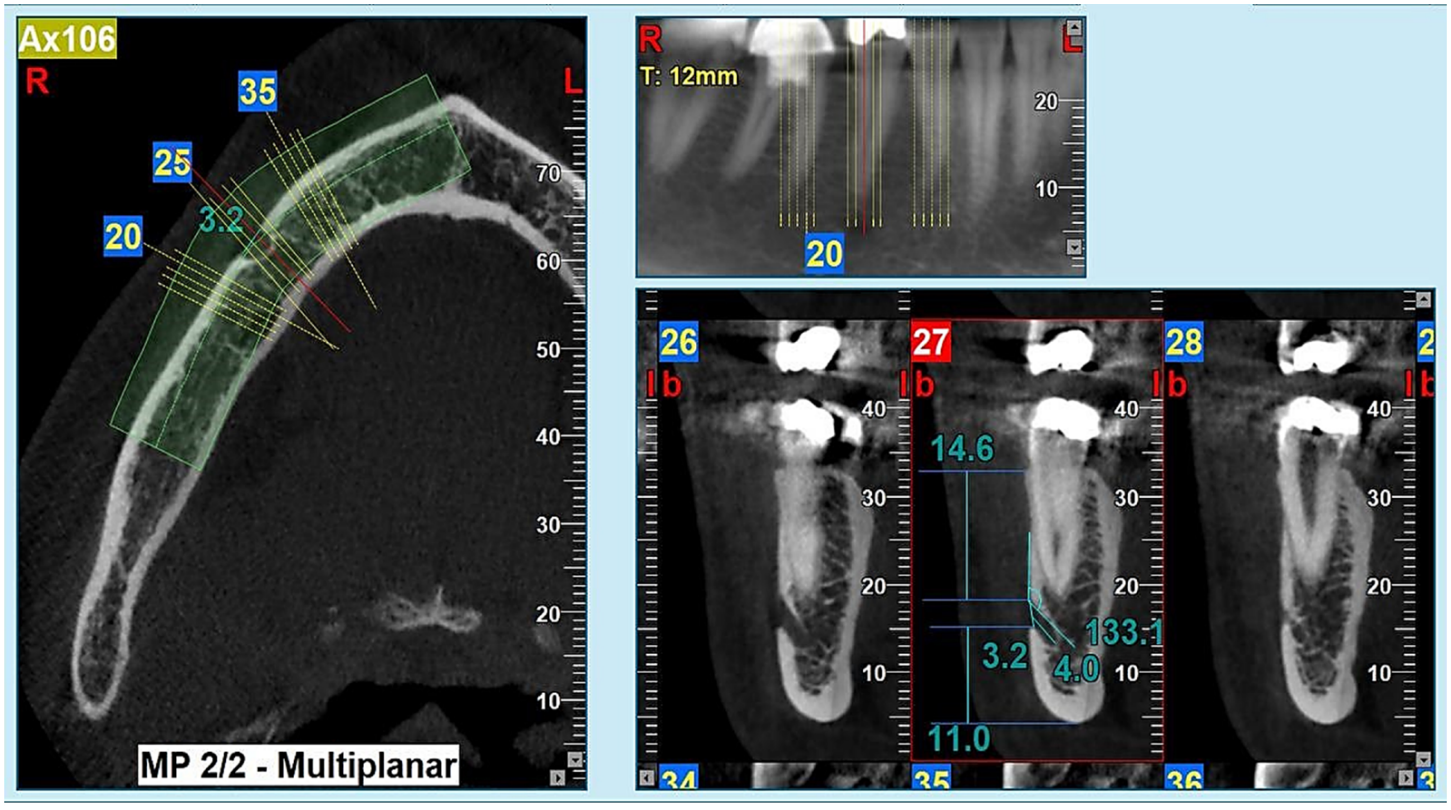
Assessment of mental foramen and canal using cone-beam computed tomography imaging technique.

The investigated parameters, as shown in Figures 1 and 2, encompassed the followings:

1. The angle of the mental canal: The angle and orientation of the mental canal relative to the buccal plate of the mandible bone were measured in the cross-sectional sections.

2. The length of the mental canal: This parameter involved measuring the distance between the mental foramen opening and the canal of the teeth in the cross-sectional view.

3. The direction of the opening of the mental canal: In the axial plane, the opening of the mental canal was assessed as either mesial, distal, or directly oriented.

4. The vertical diameter of the mental foramen: In cross-sectional slices, the distance between the apex of the upper and lower edges of the mental foramen cortical bone was measured diagonally.

5. The width of the mental foramen: This parameter entailed measuring the distance between the mesial and distal margins of the mental foramen, as observed in axial slices.

6. The distance of the mental foramen to the upper edge of the alveolar crest: In the cross-sectional view, a horizontal line parallel to the lower edge of the mandible cortex was drawn from the upper edge of the canal. The distance between this line and the crest was then measured.

7. The distance of mental foramen to the lower border of the mandible: In the cross-sectional view, a horizontal line parallel to the alveolar crest was drawn from the lower edge of the mandible canal. The distance between this line and the lower border of the mandible was measured.

8. The space of mental foramen: We recorded the teeth or interdental spaces of the mental foramen.

9. The number of the mental foramen.

-Statistical analyses

The collected data underwent descriptive and analytical statistics. The frequency and mean of the data were estimated where necessary. The normality of the data was assessed using the Kolmogorov-Smirnov test. We also used the chi-squared test and sample t-test (in case of normal data distribution) or the Mann-Whitney U test (if the data were not normally distributed) for data analysis. The significance level was set at *p-value* <0.05, and the statistical software used for the analyses was SPSS version 22.

-Ethical approval

Before the study, we took all participants’ written consent forms. The obtained information was kept confidential, and it was forbidden to mention the names and surnames of the patients. The protocol was approved by the ethics committee of Babol University of Medical Sciences (IR.MUBABOL.REC.1401.109).

## Results

In this study, a total of 710 terminal branches of the mental foramen and canal were finally investigated (n=355 on the left mandible and n=355 on the right mandible). Overall, 47.9% of the subjects were men and 52.1% were women, with a mean age of 41.87±13.08 years old. The frequency of single main mental foramen was n=682 (96.1%); this rate for single mental foramen plus single accessory mental foramen was n=17 (2.4%), and for single mental foramen plus ≥two accessory mental foramina was n=11 (1.5%).

Regarding the direction of the opening of the mental canal in the axial plane, the frequencies of mesial, distal, and direct openings were respectively n=48 (13.5%), n=159 (44.8%) and n=148 (41.7%) on the left mandible, and n=60 (16.9%), n=175 (49.3%) and n=120 (33.8%) on the right mandible. According to the analysis, there was no significant difference between the right and left mandibles in the direction of the opening of the mental canal (*p*=0.081).

Concerning the mental foramen space, the frequencies of teeth and interdental spaces were respectively n=123 (34.6%) and n=232 (65.4%) on the left mandible, and n=136 (38.3%) and n=219 (61.7%) on the right mandible. The analysis showed that the difference between the right and left mandibles in the mental foramen space was not statistically significant (*p*=0.175).

 Table 1 represents the morphological characteristics of all mental foramina and canals studied. Overall, the mean angle of the mental canals was 135.38±18.71° (range, 76.12-179.32°), the mean length of the mental canals was 6.75±1.93 mm (range, 2.39-12.42 mm), the mean vertical diameter of the mental foramina was 4.00±1.59 mm (range, 1.36-9.28 mm), the mean width of the mental foramina was 4.26±1.31 mm (range, 1.47-7.93 mm), the mean distance between the mental foramina and the upper edge of the alveolar crests was 11.28±3.77 mm (range, 2.34-19.68 mm), and the mean distance between the mental foramina and the lower borders of the mandible was 13.59±3.81 mm (range, 8.59-18.63 mm). Comparing these data showed that there were significant differences between the left and right mandibles in the mental foramina width (*p*=0.024) and the distance between the mental foramina and lower mandibular borders (*p*<0.001).

The morphological characteristics of mental foramina and canals are summarized by sex in  Table 2. There were significant differences between men and women in the mean vertical diameter of the mental foramina on the left mandible (*p*=0.048), the mean distance between the mental foramina and the upper edge of the alveolar crests on the right mandible (*p*=0.046), and the mean distance between the mental foramina and the lower borders of the mandible on both the left (*p*=0.002) and right mandibles (*p*=0.002).

 Table 3 summarizes the anatomical characteristics of mental foramina and canals by age. Based on the analysis of the right mandible data, the mean vertical diameter of the mental foramina was significantly larger in subjects above 45 years than in those aged 45 years or younger (*p*=0.024). No significant differences were seen between the age groups in terms of other morphological variables.

## Discussion

In the present study, we evaluated the variations in the anatomical characteristics of 710 mental foramina and canals using CBCT images. Our results showed that a single mental foramen was seen in most of the scans. Most of the mental canals had a distal opening, followed by direct and mesial openings, with no significant differences between the right and left mandibles in this regard. These findings were consistent with previous studies (11-13).

We observed that the mental canal angle did not statistically differ by sex, age, and side of the mandible, which was consistent with the studies by von Arx *et al*. (14) and Abu-Ta’a *et al*. (15) but contrary to the results found by Ahmed *et al*. (16). Evaluating the mental canal angle can aid in dental implant planning, preventing nerve injury, selecting appropriate implant length and angulation, and achieving optimal prosthetic outcomes. Furthermore, it assists in diagnosing and treating mandibular fractures, tumors, and cysts (14,16,17). By utilizing this anatomical information, clinicians can enhance treatment precision, improve patient safety, and optimize overall patient care.

Similar to the mental canal angle, data analysis revealed no statistically significant differences in the length of the mental canal with respect to sex, age, and mandibular side, which was in agreement with previous findings (14,18,19). The length of the mental canal can influence implant placement, nerve-related procedures (such as inferior alveolar nerve block or nerve repositioning), diagnosis of pathology (such as mandibular fractures, tumors or cysts), and orthodontic treatment planning (19-21). Accurate knowledge of the mental canal length enables clinicians to make informed decisions, minimize potential complications, and provide optimal patient care. Therefore, this measurement holds great clinical significance and should be carefully considered in dental and maxillofacial practice.

The vertical diameter of the mental foramen was not significantly different between the left and right mandibles. However, on the left mandible, the diameter was significantly larger in men versus women. Moreover, on the right mandible, those aged >45 years had a larger diameter than those aged ≤45. In their study, Muinelo-Lorenzo *et al*. (22) reported that the mental foramen diameter was larger in men than in women; however, they did not find a significant correlation between the diameter and age. Consideration of the mental foramen diameter is crucial during local anesthesia administration to ensure effective pain control and avoid nerve injury. Moreover, in endodontic procedures, understanding the vertical diameter helps in evaluating the proximity of the mental foramen to the affected tooth, enabling appropriate treatment planning and minimizing the potential for nerve involvement (15,22,23).

According to our analyses, the width of the mental foramen was larger on the right mandible compared with the left mandible. There were no significant differences in the mental foramen width on the left and right mandibles by sex and age. Abu-Ta’a *et al*. (15) stated that the width of the mental foramen did not significantly differ by the mandibular side, which was inconsistent with our results. They also reported that it was larger in men than in women, which was contrary to our findings. On the other hand, the mental foramen width was not stated to be affected by age, which aligns with our results. Moreover, Fontenele *et al*. (24) mentioned that the mental foramen width was not significantly different between men and women, which was consistent with the current study. The width of the mental foramen can guide the administration of local anesthesia, aids in dental implant placement, helps prevent nerve injury, assists in the diagnosis and treatment of mandibular pathologies, and ensures overall patient safety and comfort (24,25).

In this study, the distance between the mental foramen and upper alveolar crest edge did not significantly differ by mandibular side and age. On the other hand, this parameter was greater in men versus women on the right mandible. We also found that the distance between the mental foramen and the lower mandibular border was significantly greater on the left mandible than on the right mandible. It was also greater in men compared with women on both right and left mandibles. Conversely, the distance did not significantly differ by age. Accurate measurements of these distances help in implant placement, nerve block injections (e.g., inferior alveolar nerve block), and surgical interventions (e.g., orthognathic surgery or osteotomies), ensuring the safety and success of the treatments (14-16). It is important to emphasize that these measurements should not be examined in isolation but as an integral part of a comprehensive treatment plan.

This study had some limitations that should be acknowledged. These include the limited sample representing a specific region, potential selection bias due to the retrospective design, and the lack of exploration into the clinical implications of the anatomical variations observed. The potential operator variability in analyzing CBCT images could further impact the accuracy of the findings. Additionally, the single assessment of the study of CBCT scans limits the understanding of longitudinal changes. Further research is needed to address these limitations and determine the effect of radiographic exposure parameters on interpreting the visibility of mental foramen borders.

## Conclusions

According to the results, differences between the left and right mandibles, as well as sex- and age-related differences, were observed in the morphological characteristics of the mental foramen and canal. Overall, these findings provided valuable insights into the anatomical variations of the mental foramen and canal, which have implications for oral and maxillofacial procedures and pathologies. Further studies are needed to validate and expand upon these observations.

## Figures and Tables

**Table 1:** Morphological characteristics of mental foramina and canals in right (n=355) and left (n=355) mandibles.

Variable	Side of mandible	Mean±SD	P-value
Mental canal angle (degree)	Left	134.26±18.39	0.111
	Right	136.50±18.98	
	Overall	135.38±18.71	-
Mental canal length (mm)	Left	6.62±1.89	0.074
	Right	6.88±1.96	
	Overall	6.75±1.93	-
Mental foramen vertical diameter (mm)	Left	3.89±1.59	0.076
	Right	4.10±1.58	
	Overall	4.00±1.59	-
Mental foramen width (mm)	Left	4.15±1.31	0.024*
	Right	4.37±1.30	
	Overall	4.26±1.31	-
Distance between mental foramen and upper alveolar crest edge (mm)	Left	11.31±3.67	0.836
	Right	11.25±3.88	
	Overall	11.28±3.77	-
Distance between mental foramen and lower mandibular border (mm)	Left	13.92±3.73	<0.001*
	Right	12.25±3.94	
	Overall	13.59±3.81	-

**Table 2:** Morphological characteristics of mental foramina and canals by sex (men, n=170; women, n=185).

	Variable	Sex	Mean±SD	P-value
Left mandible	Mental canal angle (degree)	Men	132.55±19.60	0.091
		Women	135.85±17.10	
	Mental canal length (mm)	Men	6.75±1.97	0.210
		Women	6.50±1.81	
	Mental foramen vertical diameter (mm)	Men	4.07±1.71	0.048*
		Women	3.73±1.48	
	Mental foramen width (mm)	Men	4.22±1.30	0.298
		Women	4.08±1.33	
	Distance between mental foramen and upper alveolar crest edge (mm)	Men	11.35±3.77	0.864
		Women	11.28±3.57	
	Distance between mental foramen and lower mandibular border (mm)	Men	14.12±3.95	0.002*
		Women	12.83±3.64	
Right mandible	Mental canal angle (degree)	Men	135.17±19.59	0.206
		Women	137.72±18.38	
	Mental canal length (mm)	Men	7.02±2.03	0.209
		Women	6.75±1.88	
	Mental foramen vertical diameter (mm)	Men	4.27±1.66	0.054
		Women	3.95±1.48	
	Mental foramen width (mm)	Men	4.43±1.35	0.367
		Women	4.31±1.24	
	Distance between mental foramen and upper alveolar crest edge (mm)	Men	11.68±3.72	0.046*
		Women	10.86±3.98	
	Distance between mental foramen and lower mandibular border (mm)	Men	13.72±3.81	0.002*
		Women	12.46±3.83	

**Table 3:** Morphological characteristics of mental foramina and canals by age (≤45 years, n=214; >45 years, n=141).

	Variable	Age (years)	Mean±SD	P-value
Left mandible	Mental canal angle (degree)	≤45	135.71±18.59	0.068
		>45	132.07±17.93	
	Mental canal length (mm)	≤45	6.71±1.80	0.267
		>45	6.48±2.02	
	Mental foramen vertical diameter (mm)	≤45	3.89±1.67	0.917
		>45	3.90±1.47	
	Mental foramen width (mm)	≤45	4.20±1.32	0.360
		>45	4.07±1.30	
	Distance between mental foramen and upper alveolar crest edge (mm)	≤45	11.29±3.65	0.869
		>45	11.35±3.70	
	Distance between mental foramen and lower mandibular border (mm)	≤45	14.29±3.87	0.175
		>45	13.73±3.68	
Right mandible	Mental canal angle (degree)	≤45	135.92±17.99	0.479
		>45	137.38±20.43	
	Mental canal length (mm)	≤45	7.02±2.07	0.102
		>45	6.67±1.75	
	Mental foramen vertical diameter (mm)	≤45	3.95±1.46	0.024*
		>45	4.34±1.73	
	Mental foramen width (mm)	≤45	4.70±1.27	0.544
		>45	4.32±1.34	
	Distance between mental foramen and upper alveolar crest edge (mm)	≤45	11.35±3.71	0.564
		>45	11.11±4.13	
	Distance between mental foramen and lower mandibular border (mm)	≤45	13.37±3.05	0.408
		>45	13.07±3.73	

## Data Availability

The datasets used and/or analyzed during the current study are available from the corresponding author.
